# Effect of Past Chlamydophila pneumoniae Infection on the Short-Time Mortality of COVID-19: A Retrospective Cohort Study

**DOI:** 10.7759/cureus.34543

**Published:** 2023-02-02

**Authors:** Hiroshi Horiuchi, Syusuke Utada, Yoshie Shinomiya, Azusa Sogo, Takao Miyagawa, Shoko Niida, Hiromu Okano, Naoya Suzuki, Tsuyoshi Otsuka, Hiroshi Miyazaki, Ryosuke Furuya

**Affiliations:** 1 Department of Critical Care and Emergency Medicine, National Hospital Organization Yokohama Medical Center, Yokohama, JPN

**Keywords:** antibody, retrospective cohort study, persistent infection, vascular disease, chlamydophila pneumoniae, covid-19

## Abstract

Background: Although *Chlamydophila pneumoniae *(CP)is known to play a role in atherosclerosis and endothelial injury, its past infection on the mortality of coronavirus disease 2019 (COVID-19), which was also reported to be a vascular disease, remains unknown.

Methods: In this retrospective cohort study, we examined 78 COVID-19 patients and 32 bacterial pneumonia patients who visited a tertiary emergency center in Japan between April 1, 2021, and April 30, 2022. CP antibody levels, including IgM, IgG, and IgA, were measured.

Results: Among all patients, the CP IgA-positive rate was significantly associated with age (P = 0.002). Between the COVID-19 and non-COVID-19 groups, no difference in the positive rate for both CP IgG and IgA was observed (P = 1.00 and 0.51, respectively). The mean age and proportion of males were significantly higher in the IgA-positive group than in the IgA-negative group (60.7 vs. 75.5, P = 0.001; 61.5% vs. 85.0%, P = 0.019, respectively). Smoking and dead outcomes were significantly higher both in the IgA-positive group and IgG-positive group (smoking: 26.7% vs. 62.2, P = 0.003; 34.7% vs. 73.1%, P = 0.002, dead outcome: 6.5% vs. 29.8%, P = 0.020; 13.5% vs. 34.6%, P = 0.039, respectively). Although the log-rank test revealed higher 30-day mortality in the IgG-positive group compared to the IgG-negative group (P = 0.032), Cox regression analysis demonstrated no significant difference between the IgG-positive and negative groups (hazard ratio (HR) = 4.10, 95%CI = 0.94-18.0, P = 0.061).

Conclusion: The effect of past CP infection on 30-day mortality in COVID-19 patients was not obvious.

## Introduction

*Chlamydophila pneumoniae* (CP) is known to play a role in atherosclerosis, especially of the coronary artery, by disseminating from the lung to extrapulmonary tissues, such as vascular walls [1,2]. Although CP is thought to be one of the major pathogens causing community-acquired pneumonia in Japan as well as in other countries [3], several studies have shown that its prevalence in Japan has been quite low [4,5]. Severe cardiovascular complications have been reported in patients with coronavirus disease 2019 (COVID-19) [6], and COVID-19 itself is reported to be a vascular disease that causes endothelial injury leading to multiple organ dysfunction [7].

Although CP coinfection rates in COVID-19 patients were reported to be as high as 18% in Italy and 29.1-36.4% in Nigeria [8,9], those in China, Brazil, and India were reported to be 0.3%, 0.0%, and 3.6%, respectively [10-12]. CP coinfection has been reported to aggravate the clinical course of COVID-19 [12].

Lifestyle-related diseases related to atherosclerosis and endothelial injury, such as diabetes mellitus, hypertension, and dyslipidemia, have been reported to worsen the prognosis of COVID-19 [13-15]. Although the prognosis of COVID-19 is not solely due to the advancement of coronary artery disease, it is possible that previous CP infection affects the clinical course of COVID-19 due to its effect of atherosclerosis and endothelial injury to other vessels. However, It is unknown whether past CP infection affects the mortality of COVID-19 owing partly to atherosclerosis and endothelial injury. Therefore, this study aimed to investigate whether previous CP infection affects the mortality of COVID-19.

This article was previously posted on the Research Square preprint server on September 7, 2022.

## Materials and methods

Study population

This retrospective cohort study was performed at a tertiary emergency center. COVID-19 patients with COVID pneumonia and non-COVID-19 patients with bacterial pneumonia who were hospitalized at the National Hospital Organization Yokohama Medical Center between April 1, 2021, and April 30, 2022, regardless of severity, and whose CP antibody levels were measured at least once at the time of admission, were included. COVID-19 patients without pneumonia were not included. COVID-19 was diagnosed based on a positive severe acute respiratory syndrome coronavirus 2 (SARS-CoV-2) polymerase chain reaction test result. Age, sex, ethnicity, body mass index (BMI), smoking history, vaccination against COVID-19 (VAC), history of HTN, DM, DL, oxygen demand at the time of admission (O2), and clinical outcome were recorded. O2 was categorized into mild (no oxygen administration, oxygen administration using a nasal cannula or a face mask) or severe (oxygen administration using a reservoir mask, a high-flow nasal cannula, or mechanical ventilator). The clinical outcome was categorized as either alive or dead on day 30 after hospitalization.

Serological testing

CP antibodies, including IgM, IgG, and IgA, were measured using an ELNAS Plate CP commercial test kit. CP antibodies were measured at least once, and for some patients, were measured twice at 14-day intervals. The first antibody titers were used for statistical evaluation. Age-related prevalence of CP antibody positivity rates was investigated by dividing age by 10 years. CP antibody positivity was defined as each CP antibody value above the cutoff values. The cutoff values for CP IgM, IgG, and IgA were 1.10, 30, and 8, respectively. Differences in CP antibody positivity rates between the COVID-19 and non-COVID-19 groups were studied. Background data at the time of admission and mortality rate were compared between antibody carriers and non-carriers of both IgG and IgA.

Statistical analysis

We used Spearman’s rank correlation test to determine the relationship between age divided by 10 years and CP antibody (IgG and IgA) positivity rate in both COVID-19 and non-COVID-19 groups. We used Chi-square tests to compare the proportions of categorical variables (such as sex), and t-tests to compare differences in mean age and BMI between groups. Log-rank test and Cox regression analysis were performed to compare the mortality between CP antibody-positive groups and CP antibody-negative groups. The threshold for significance was set at P < 0.05. We used the statistical software EZR version 1.55 for all analyses [16].

## Results

Seventy-eight COVID-19 patients with pneumonia and 32 non-COVID-19 patients with bacterial pneumonia were included. None of the 110 patients had positive IgM results. The second measurement of CP antibody levels in 10 COVID-19 patients and two non-COVID-19 patients was performed after a 14-day interval. In only one COVID-19 patient, both CP IgG and IgA levels increased after this interval (See Appendix).

**Figure 1:**
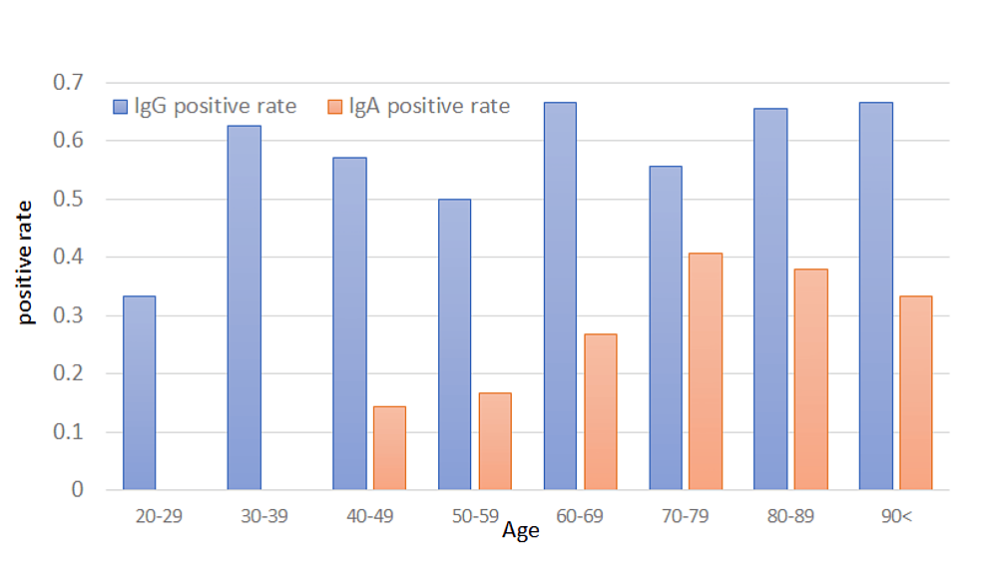
Relationship between age and CP antibody positivity rate in all patients studied. P-value was 0.09 for CP IgG, and 0.002 for CP IgA by Spearman’s rank correlation test CP, *Chlamydophia pneumoniae*

In this study, age was significantly associated with CP IgA-positivity rate (P = 0.002) but not with CP IgG-positivity rate (P = 0.09). None of the patients aged < 47 years had positive CP IgA levels (Figure 1).

Between the COVID-19 and non-COVID-19 groups, no difference in the CP IgG- and IgA-positivity rates was observed (P = 1.00, and 0.51, respectively) (Table 1).

**Table 1:** Difference of positive rate for CP IgG and IgA between COVID-19 and non-COVID-19 patients CP, *Chlamydophila pneumoniae; *COVID-19, coronavirus disease 2019

	COVID-19	non-COVID-19	p.value
IgG positive (%)	47 (60.3)	19 (59.4)	1.00
IgA positive (%)	25 (33.3)	13 (40.6)	0.51

The mean age and proportion of males were significantly higher in the IgA-positive group than in the IgA-negative group. Smoking and dead outcomes were significantly higher both in the IgA-positive group and IgG-positive group (Table 2).

**Table 2:** Background characteristics of CP IgG and IgA positive and negative groups BMI, body mass index; VAC, vaccination against COVID-19; HTN, hypertension; DM, diabetes mellitus; DL, dyslipidemia; O2, oxygen demand at the time of admission; CP, *Chlamydophila pneumoniae*

	IgG - (n=31)	IgG + (n=47)	p.value		IgA - (n=52)	IgA + (n=26)	p.value
Age (range)	62.0 (26-99)	67.7 (28-94)	0.18		60.7 (26-99)	75.5 (52-92)	0.001
Sex, male	20 (64.5)	35 (74.5)	0.32		32 (61.5)	22 (85.0)	0.019
Ethnicity, Asian (%)	30 (96.8)	48 (100)	1.00		51 (98.1)	26 (100)	1.00
Ethnicity, Caucasian (%)	1 (3.2)	0 (0.0)	1.00		1 (1.9)	0 (0.0)	1.00
BMI (range)	26.1(17.8-74.8)	23.7 (13.8-35.4)	0.18		25.7 (17.8-74.8)	22.5 (13.8-29.9)	0.089
Smoking (%)	8 (26.7)	28 (62.2)	0.003		17 (34.7)	19 (73.1)	0.002
VAC (%)	10 (32.2)	18 (38.3)	0.77		15 (28.8)	13 (50.0)	0.21
HTN (%)	12 (38.7)	25 (53.2)	0.17		23 (44.2)	14 (53.4)	0.63
DM (%)	12 (38.7)	18 (38.3)	1.00		19 (36.5)	11 (42.3)	0.81
DL (%)	5 (16.1)	14 (29.8)	0.28		13 (25.0)	6 (23.1)	1.00
O2, severe (%)	13 (41.9)	17 (36.2)	0.63		21 (40.4)	9 (34.6)	0.81
Outcome, dead (%)	2 (6.5)	14 (29.8)	0.020		7 (13.5)	9 (34.6)	0.039

**Figure 2:**
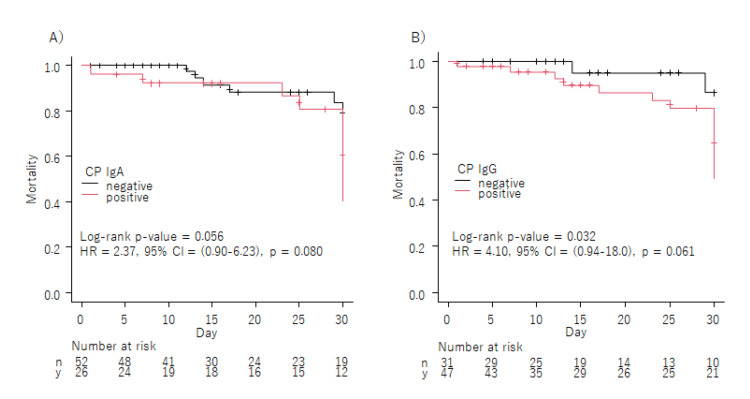
Log-rank test and Cox regression analysis for CP IgA-positive and IgA-negative groups (A), and CP IgG-positive and IgG-negative groups (B) CP, *Chlamydophila pneumoniae; *HR, hazard ratio

Although the log-rank test revealed higher 30-day mortality only in the IgG-positive group (Figure 2), Cox regression analysis demonstrated no significant difference between the IgG-positive and negative groups.

## Discussion

This study investigated the association between past CP infection and the clinical course of COVID-19 inpatients with pneumonia at a tertiary emergency center. The results showed that the CP IgA-positive rate was significantly associated with age in all patients studied. Although the dead outcome was significantly higher both in the IgA-positive group and IgG-positive group, and the log-rank test revealed higher 30-day mortality in the IgG-positive group compared to the IgG-negative group, Cox regression analysis demonstrated no significant difference between the IgG-positive and negative groups.

Previous studies have shown that the CP coinfection rate in COVID-19 patients was high in several countries other than Japan and aggravated the clinical course of COVID-19. Among our patients studied, no patients were positive for CP IgM, and positive rates of both CP IgG and IgA were not significantly different between the COVID-19 and non-COVID-19 groups (Table 1). This study suggested that acute CP coinfection in COVID-19 patients in Japan was rare and CP coinfection was not especially higher in COVID-19 patients, at least in the area near our hospital. To the best of our knowledge, no other study has investigated the relationship between past CP infections and COVID-19 mortality. The current study suggests that past CP infection may have no effect on the clinical course of COVID-19.

The relationship between age and IgA positivity only supports the findings of past studies that CP IgA positivity suggests chronic CP infection. But our study could not show the higher 30-day mortality rate in the CP IgA-positive group (Figure 2).

It was biologically plausible that the positivity of CP IgA, which may indicate chronic CP infection, was related to a higher mortality rate of COVID-19 patients through arteriosclerosis and endothelial injury, although it was possible that higher mortality in the CP IgA-positive group was confounded by older age, a higher proportion of males, and smoking. A female advantage in COVID-19 has previously been reported by a global meta-analysis on COVID-19 considering both the innate and adaptive immune systems [17]. A previous study in Japan reported that the prevalence of CP IgG in elderly individuals (≥ 60 years) was significantly higher in men than in women (75.5% vs. 64.8%; P = 0.00029) [3]. Although this sex-related difference in antibody positivity was almost consistent with our study, our study indicated that CP IgA was only detectable in patients older than 40 years. CP IgA is considered a marker of immune response and local inflammation in arterial walls induced by CP [18]. Thus, CP IgA, but not CP IgG, was considered a putative marker of chronic infection and subsequent risk of death from not only ischemic heart disease by rupture of atheromatous plaques but also multiple organ dysfunction by endothelial injury. Similarities in the inflammatory processes operating in COVID-19 and atherosclerosis have been suggested [6], and it is possible that arteriosclerosis and endothelial injury due to past CP infection affect the prognosis of COVID-19. The prognosis is worse in older men with COVID-19. In general, older men are immunologically disadvantaged against infection, which may explain the poorer prognosis of older men with COVID-19 and the higher number of CP IgA-positive cases, suggesting persistent CP infection in older men. Although our study did not show direct evidence of persistent infection with CP, persistent infection with CP in older men may worsen the prognosis of COVID-19 via vascular lesions. A relationship between smoking and CP-antibody positivity has been reported [19,20]. Damaged airways and impaired immune response as a result of smoking were considered as the reasons [19]. In CP-antibody-positive patients, smoking was reported to be a risk for coronary artery calcification progression [21]. Our study could not show statistical significance between positive CP serology and mortality. Although multivariate analysis could not be attempted due to the small number of dead cases, older age, male, and smoking history have already been known to be associated with worse prognosis in COVID-19 [22,23], and it is likely that these factors were confounding factors that increased mortality in the CP antibody-positive cases.

This study has several limitations. First, it was a single-center cohort study with a limited number of cases including several variants of SARS-CoV-2. Mortality of COVID-19 cases was highly dependent on its variant types and patient vaccination backgrounds. Second, a comparison of mortality with non-COVID-19 pneumonia could not be performed because none of the non-COVID-19 cases died. Third, biases related to arteriosclerosis due to unobserved subjects can affect the clinical course of COVID-19 patients with past CP infections.

## Conclusions

We performed a retrospective cohort study of COVID-19 patients in a tertiary emergency center to investigate the effect of past CP infection on the mortality of the disease. Although it was biologically plausible that CP IgA-positive COVID-19 patients had a poorer prognosis, no statistical difference in 30-day mortality was observed in either CP IgA-positive or CP IgG-positive COVID-19 patients. Further large-scale studies are needed to evaluate the effect of past CP infection on COVID-19 short-time mortality.

## Appendices

**Table 3:** Raw data of patient characteristics BMI, body mass index; COVID-19, coronavirus 2019; VAC, vaccination against COVID-19; HTN, hypertension; DM, diabetes mellitus; DL, dyslipidemia; O2, oxygen demand at the time of admission; IgM2, *Chlamydophila pneumoniae* IgM two weeks after the initial measurement; IgG2, *Chlamydophila pneumoniae *IgG two weeks after the initial measurement; IgA2, *Chlamydophila pneumoniae* IgA two weeks after the initial measurement; NC, nasal cannula; FM, face mask; RM, reservoir mask; HFNC, high flow nasal cannula; y, yes; n, no; M, male; F, female; NA, not available; EIU, enzyme immunoassay units

Age	Sex	Ethnicity	COVID-19	BMI (kg/m^2^)	Smoking	VAC	DM	HTN	DL	O2	Follow-up period (days)	Outcome	IgM	IgA (EIU)	IgG (EIU)	IgM2	IgA2	IgG2
78	F	Asian	y	13.8	y	n	n	y	n	NC 2l	8	alive	0	8	147	NA	NA	NA
89	M	Asian	y	15.8	y	y	n	y	n	FM 9l	32	dead	0	43	126	NA	NA	NA
90	M	Asian	y	16.0	y	y	n	y	n	HFNC	7	dead	0	12	84	NA	NA	NA
92	M	Asian	y	16.2	n	y	n	y	n	HFNC	196	alive	0	53	155	NA	NA	NA
99	F	Asian	y	17.8	n	y	n	y	n	NC 4l	18	alive	0	1	24	NA	NA	NA
85	M	Asian	y	17.9	y	NA	y	y	y	none	13	alive	0	5	40	NA	NA	NA
81	F	Asian	y	18.4	n	n	n	n	n	Ventilator	24	alive	0	0	17	NA	NA	NA
80	F	Asian	y	18.6	n	NA	n	y	n	none	11	alive	0	1	14	NA	NA	NA
81	M	Asian	y	18.6	n	n	n	n	n	NC 2l	194	alive	NA	53	99	NA	NA	NA
72	M	Asian	y	18.9	y	n	n	y	y	HFNC	5	alive	0	7	34	NA	NA	NA
92	F	Asian	y	19.2	NA	y	n	y	n	none	214	alive	0	3	52	NA	NA	NA
83	M	Asian	y	19.4	y	n	y	n	n	NC 4l	154	alive	0	12	49	NA	NA	NA
70	M	Asian	y	19.8	y	y	n	n	n	none	5	alive	0	3	22	NA	NA	NA
88	M	Asian	y	20.0	n	y	y	n	n	HFNC	9	alive	0	21	131	NA	NA	NA
77	M	Asian	y	20.2	y	y	n	y	n	NC 1l	31	alive	0	20	180	NA	NA	NA
88	M	Asian	y	20.3	y	y	y	y	n	none	115	alive	0	17	36	NA	NA	NA
57	M	Asian	y	20.4	y	n	y	y	y	Ventilator	194	alive	0	94	191	0	73	165
71	M	Asian	y	20.7	y	y	y	y	y	Ventilator	16	alive	0	6	16	NA	NA	NA
74	F	Asian	y	21.0	n	y	y	y	n	Ventilator	28	alive	0	6	24	NA	NA	NA
60	M	Asian	y	21.4	y	y	n	n	y	none	154	alive	0	2	60	NA	NA	NA
62	F	Asian	y	21.4	n	n	y	n	n	HFNC	355	alive	0	5	43	NA	5	32
76	M	Asian	y	21.4	y	y	y	y	y	HFNC	2	dead	0	5	69	NA	NA	NA
76	M	Asian	y	21.7	NA	y	n	n	n	none	17	alive	0	16	136	NA	NA	NA
71	F	Asian	y	21.9	n	y	n	y	y	Ventilator	15	alive	0	1	15	NA	NA	NA
59	M	Asian	y	22.0	y	n	y	n	n	Ventilator	7	alive	0	0	3	NA	NA	NA
81	M	Asian	y	22.0	n	n	n	y	n	HFNC	85	alive	0	7	47	NA	NA	NA
62	M	Asian	y	22.1	y	n	y	n	n	NC 4l	39	dead	0	11	146	NA	NA	NA
33	F	Asian	y	22.5	y	n	n	n	n	NC 4l	25	alive	0	6	94	NA	NA	NA
63	F	Asian	y	22.6	y	n	n	n	n	NC 4l	11	alive	0	3	2	NA	NA	NA
70	M	Asian	y	22.6	y	y	n	n	n	HFNC	83	alive	0	15	23	NA	NA	NA
51	F	Asian	y	22.8	n	n	n	n	n	NC 1l	7	alive	0	2	29	NA	NA	NA
62	F	Asian	y	22.8	n	n	n	n	n	NC 1l	12	alive	0	1	11	NA	NA	NA
72	F	Asian	y	22.8	n	n	n	n	y	NC 1l	18	alive	0	6	65	NA	5	53
90	M	Asian	y	22.9	n	y	y	y	n	HFNC	427	dead	0	14	44	NA	NA	NA
68	M	Asian	y	23.0	y	n	n	n	n	Ventilator	30	alive	0	0	3	NA	NA	NA
94	F	Asian	y	23.3	n	y	y	y	n	NC 4l	10	alive	0	4	75	NA	NA	NA
88	M	Asian	y	23.4	n	n	y	y	y	NC 2l	38	alive	0	97	69	NA	NA	NA
53	M	Asian	y	23.5	y	n	n	n	n	NC 1l	14	alive	0	1	48	NA	NA	NA
75	M	Asian	y	23.5	n	n	n	n	n	NC 2l	25	alive	0	0	1	NA	NA	NA
76	M	Asian	y	23.5	y	NA	n	n	n	NC 3l	392	alive	0	17	275	NA	NA	NA
84	M	Asian	y	23.9	n	n	n	y	n	HFNC	8	dead	0	3	20	NA	NA	NA
41	F	Asian	y	24.1	n	n	n	n	y	NC 1l	14	alive	0	0	5	NA	NA	NA
71	M	Asian	y	25.0	y	n	n	y	n	Ventilator	17	dead	0	26	185	NA	NA	NA
81	F	Asian	y	25.2	n	n	y	y	y	Ventilator	23	alive	0	2	18	NA	NA	NA
44	M	Asian	y	25.3	y	n	y	n	n	Ventilator	140	alive	0	3	34	0	2	30
62	M	Asian	y	25.5	y	n	n	y	y	FM 6l	30	dead	0	9	135	0	7	82
65	M	Asian	y	25.5	n	y	n	y	y	HFNC	43	alive	0	6	44	NA	NA	NA
56	M	Asian	y	25.8	n	n	y	y	n	Ventilator	33	alive	0	5	26	0	8	15
64	M	Asian	y	26.0	n	y	n	y	n	Ventilator	368	alive	0	3	40	NA	NA	NA
59	M	Asian	y	26.3	y	n	y	n	n	NC 2l	8	alive	0	1	34	NA	0	31
75	M	Asian	y	26.4	y	n	n	n	n	Ventilator	319	dead	0	15	43	NA	NA	NA
65	F	Asian	y	26.5	y	n	y	n	y	Ventilator	1	dead	0	8	86	NA	12	130
34	F	Asian	y	26.7	y	n	n	n	n	NC 1l	46	alive	0	4	56	NA	NA	NA
40	M	Asian	y	26.7	n	n	n	y	n	none	6	alive	0	2	27	NA	NA	NA
52	M	Caucasian	y	26.7	n	n	n	n	n	NC 1l	4	alive	0	0	0	NA	NA	NA
39	F	Asian	y	26.8	y	n	n	n	n	NC 2l	4	alive	0	3	66	NA	NA	NA
28	M	Asian	y	28.0	n	y	n	y	n	NC 4l	11	alive	0	5	73	NA	NA	NA
38	F	Asian	y	28.7	n	n	y	n	n	HFNC	9	alive	0	1	0	NA	NA	NA
57	M	Asian	y	28.7	y	y	n	n	y	NC 3l	239	alive	0	18	107	NA	NA	NA
65	M	Asian	y	28.7	y	y	y	n	y	none	16	alive	0	8	102	NA	NA	NA
63	M	Asian	y	29.0	n	n	y	n	n	NC 2l	4	alive	0	2	29	NA	NA	NA
53	M	Asian	y	29.7	n	n	n	n	n	Ventilator	13	dead	NA	1	13	NA	NA	NA
67	F	Asian	y	29.9	n	n	n	n	n	NC 1l	29	alive	NA	19	144	NA	NA	NA
53	M	Asian	y	30.1	n	n	n	y	y	NC 2l	397	alive	NA	2	69	NA	NA	NA
26	M	Asian	y	31.6	n	y	y	n	n	none	13	alive	0	3	29	NA	NA	NA
81	M	Asian	y	31.6	y	n	y	y	n	FM 8l	16	dead	NA	5	47	NA	NA	NA
52	M	Asian	y	31.8	y	n	y	y	n	NC 4l	30	alive	NA	9	55	NA	NA	NA
38	M	Asian	y	33.1	n	n	n	n	n	NC 2l	83	alive	0	0	13	NA	NA	NA
49	F	Asian	y	33.7	n	n	y	n	n	Ventilator	10	alive	0	4	173	NA	5	85
34	M	Asian	y	35.4	n	n	n	y	y	HFNC	104	dead	0	4	82	0	9	50
47	M	Asian	y	35.7	n	n	y	y	y	none	12	alive	0	6	23	NA	NA	NA
26	M	Asian	y	74.8	n	n	y	n	n	NC 3l	26	alive	0	1	21	NA	NA	NA
34	M	Asian	n	NA	NA	NA	n	n	n	Ventilator	NA	alive	NA	3	18	NA	NA	NA
35	F	Asian	y	NA	n	n	n	n	n	none	1	alive	0	1	31	NA	NA	NA
48	F	Asian	n	NA	NA	NA	n	n	n	HFNC	NA	alive	0	10	83	NA	14	76
49	F	Asian	n	NA	NA	NA	n	n	n	FM 5l	NA	alive	0	4	50	NA	NA	NA
59	M	Asian	y	NA	NA	n	n	n	n	Ventilator	25	alive	NA	0	5	NA	NA	NA
64	M	Asian	y	NA	NA	n	y	y	y	Ventilator	NA	alive	NA	12	29	NA	NA	NA
65	M	Asian	n	NA	NA	NA	y	y	y	Ventilator	NA	alive	0	12	31	NA	NA	NA
71	F	Asian	n	NA	NA	NA	NA	NA	NA	HFNC	NA	alive	1	12	148	NA	NA	NA
74	M	Asian	n	NA	NA	NA	NA	NA	NA	FM 7l	NA	alive	0	10	248	NA	NA	NA
74	M	Asian	n	NA	NA	y	n	y	n	NC 4l	NA	alive	0	11	86	NA	NA	NA
74	F	Asian	n	NA	NA	NA	NA	NA	NA	none	NA	alive	0	0	2	NA	NA	NA
75	M	Asian	n	NA	NA	NA	y	n	n	NC 4l	NA	alive	0	7	12	NA	NA	NA
76	M	Asian	y	NA	NA	y	y	n	n	none	5	alive	0	3	7	NA	NA	NA
77	F	Asian	n	NA	NA	NA	n	n	n	NC 2l	NA	alive	0	12	31	0	10	105
77	F	Asian	n	NA	NA	NA	NA	NA	NA	HFNC	NA	alive	0	2	26	NA	NA	NA
77	M	Asian	n	NA	NA	NA	n	n	n	none	NA	alive	0	5	50	NA	NA	NA
78	M	Asian	n	NA	NA	NA	NA	NA	NA	HFNC	NA	alive	0	1	24	NA	NA	NA
79	M	Asian	n	NA	NA	NA	NA	NA	NA	HFNC	NA	alive	NA	56	256	NA	NA	NA
79	M	Asian	n	NA	NA	NA	NA	NA	NA	HFNC	NA	alive	0	8	17	NA	NA	NA
81	M	Asian	n	NA	NA	NA	y	y	n	none	NA	alive	0	13	104	NA	NA	NA
82	F	Asian	n	NA	NA	NA	NA	NA	NA	NC 1l	NA	alive	0	4	22	NA	NA	NA
83	M	Asian	n	NA	NA	NA	NA	NA	NA	Ventilator	NA	alive	0	22	87	NA	NA	NA
84	M	Asian	n	NA	NA	NA	NA	NA	NA	NC 1l	NA	alive	0	2	8	NA	NA	NA
84	M	Asian	n	NA	NA	NA	NA	NA	NA	none	NA	alive	0	9	69	NA	NA	NA
85	M	Asian	y	NA	NA	y	n	y	n	none	25	alive	0	1	9	NA	NA	NA
85	M	Asian	n	NA	NA	NA	NA	NA	NA	HFNC	NA	alive	0	3	13	NA	NA	NA
86	F	Asian	n	NA	NA	NA	NA	NA	NA	NC 1l	NA	alive	0	7	10	NA	NA	NA
86	F	Asian	n	NA	NA	NA	NA	NA	NA	NC 3l	NA	alive	NA	33	57	NA	NA	NA
87	M	Asian	n	NA	NA	NA	n	y	y	NC 2l	NA	alive	0	30	74	NA	NA	NA
87	F	Asian	n	NA	NA	NA	y	y	y	RM 12l	NA	alive	0	7	19	NA	NA	NA
88	M	Asian	y	NA	NA	y	n	y	n	none	25	alive	0	23	76	NA	NA	NA
88	F	Asian	n	NA	NA	NA	NA	NA	NA	NC 2l	NA	alive	0	4	141	NA	NA	NA
88	M	Asian	n	NA	NA	NA	n	y	n	Ventilator	NA	alive	0	7	38	NA	NA	NA
89	M	Asian	n	NA	NA	NA	n	y	n	NC 3l	NA	alive	0	6	70	NA	NA	NA
89	F	Asian	n	NA	NA	NA	n	n	n	HFNC	NA	alive	0	5	40	NA	NA	NA
90	M	Asian	n	NA	NA	NA	NA	NA	NA	NC 1l	NA	alive	0	7	32	NA	NA	NA
90	F	Asian	n	NA	NA	NA	NA	NA	NA	HFNC	NA	alive	0	6	12	NA	NA	NA
94	F	Asian	n	NA	NA	NA	NA	NA	NA	HFNC	NA	alive	0	0	8	NA	NA	NA

## References

[1] Wang SS, Tondella ML, Bajpai A (2007). Circulating Chlamydia pneumoniae DNA and advanced coronary artery disease. Int J Cardiol.

[2] Mannonen L, Markkula E, Puolakkainen M (2011). Analysis of Chlamydia pneumoniae infection in mononuclear cells by reverse transcription-PCR targeted to chlamydial gene transcripts. Med Microbiol Immunol.

[3] Miyashita N, Fukano H, Yoshida K, Niki Y, Matsushima T (2002). Seroepidemiology of Chlamydia pneumoniae in Japan between 1991 and 2000. J Clin Pathol.

[4] Noguchi S, Yatera K, Kawanami T (2017). Frequency of detection of Chlamydophila pneumoniae using bronchoalveolar lavage fluid in patients with community-onset pneumonia. Respir Investig.

[5] Fujita J, Kinjo T (2020). Where is Chlamydophila pneumoniae pneumonia?. Respir Investig.

[6] Sagris M, Theofilis P, Antonopoulos AS (2021). Inflammatory mechanisms in COVID-19 and atherosclerosis: current pharmaceutical perspectives. Int J Mol Sci.

[7] Siddiqi HK, Libby P, Ridker PM (2021). COVID-19 - a vascular disease. Trends Cardiovasc Med.

[8] De Francesco MA, Poiesi C, Gargiulo F (2021). Co-infection of chlamydia pneumoniae and mycoplasma pneumoniae with SARS-CoV-2 is associated with more severe features. J Infect.

[9] Davies-Bolorunduro OF, Fowora MA, Amoo OS (2022). Evaluation of respiratory tract bacterial co-infections in SARS-CoV-2 patients with mild or asymptomatic infection in Lagos, Nigeria. Bull Natl Res Cent.

[10] Cai F, Shou X, Ye Q (2022). Epidemiological study on Mycoplasma pneumoniae and Chlamydia pneumoniae infection of hospitalized children in a single center during the COVID-19 pandemic. Front Cell Infect Microbiol.

[11] Boschiero MN, Duarte A, Palamim CV, Alvarez AE, Mauch RM, Marson FA (2022). Frequency of respiratory pathogens other than SARS-CoV-2 detected during COVID-19 testing. Diagn Microbiol Infect Dis.

[12] Chaudhry R, Sreenath K, Batra P (2022). Atypical bacterial co-infections among patients with COVID-19: a study from India. J Med Virol.

[13] Wu Z, McGoogan JM (2020). Characteristics of and important lessons from the coronavirus disease 2019 (COVID-19) outbreak in China: summary of a report of 72 314 cases from the Chinese Center for Disease Control and Prevention. JAMA.

[14] Petrilli CM, Jones SA, Yang J (2020). Factors associated with hospital admission and critical illness among 5279 people with coronavirus disease 2019 in New York City: prospective cohort study. BMJ.

[15] Williamson EJ, Walker AJ, Bhaskaran K (2020). Factors associated with COVID-19-related death using OpenSAFELY. Nature.

[16] Kanda Y (2013). Investigation of the freely available easy-to-use software 'EZR' for medical statistics. Bone Marrow Transplant.

[17] Peckham H, de Gruijter NM, Raine C (2020). Male sex identified by global COVID-19 meta-analysis as a risk factor for death and ITU admission. Nat Commun.

[18] Strachan DP, Carrington D, Mendall MA (1999). Relation of Chlamydia pneumoniae serology to mortality and incidence of ischaemic heart disease over 13 years in the caerphilly prospective heart disease study. BMJ.

[19] Saikku P, Leinonen M, Tenkanen L (1992). Chronic Chlamydia pneumoniae infection as a risk factor for coronary heart disease in the Helsinki Heart Study. Ann Intern Med.

[20] Paltiel O, Kark JD, Leinonen M, Saikku P (1995). High prevalence of antibodies to Chlamydia pneumoniae; determinants of IgG and IgA seropositivity among Jerusalem residents. Epidemiol Infect.

[21] Player MS, Mainous AG 3rd, Everett CJ, Diaz VA, Knoll ME, Wright RU (2014). Chlamydia pneumoniae and progression of subclinical atherosclerosis. Eur J Prev Cardiol.

[22] Dessie ZG, Zewotir T (2021). Mortality-related risk factors of COVID-19: a systematic review and meta-analysis of 42 studies and 423,117 patients. BMC Infect Dis.

[23] Mahamat-Saleh Y, Fiolet T, Rebeaud ME (2021). Diabetes, hypertension, body mass index, smoking and COVID-19-related mortality: a systematic review and meta-analysis of observational studies. BMJ Open.

